# Pathways Between Family Socioeconomic Status and Early Childhood Development: Evidence From Andean Peru

**DOI:** 10.1111/desc.70260

**Published:** 2026-08-18

**Authors:** Kristen Hinckley, Dana Charles McCoy, Lena Jäggi, Stella M. Hartinger, Daniel Mäusezahl, Sarah Hatch, Milagros Alvarado, Andreana Castellanos, Leonel Aguilar, Maria Catalina Gastiaburu Cabello, Günther Fink

**Affiliations:** ^1^ Swiss Tropical and Public Health Institute Allschwil Switzerland; ^2^ University of Basel Basel Switzerland; ^3^ Universidad Peruana Cayetano Heredia Lima Peru; ^4^ Harvard Graduate School of Education Cambridge USA; ^5^ Afinidata Fort Collins USA; ^6^ Institute for Computing Platforms Federal Institute of Technology Zurich Zürich Switzerland

**Keywords:** early childhood, mediation, Peru, socioeconomic status, structural equation modelling

## Abstract

Healthy and stimulating early life environments are key for children to develop to their full potential. Household socioeconomic status (SES) is a strong predictor of early child development (ECD). This study used a rich new dataset from Andean Peru to examine the pathways through which household SES shapes ECD. Drawing on frameworks from multiple disciplines, we consider early life health, family well‐being, and family investment as mediating pathways. Factors were assessed at two time points in a sample of 2702 parent‐child dyads (49.7% children female). Measurements were conducted at infancy (*M* child age = 5.02 months) and toddlerhood (*M* child age = 29.62 months). We estimated two structural equation models to examine the direct and indirect pathways between SES and ECD. Results show that while SES predict children's health and environments in infancy and toddlerhood, the links between mediators and ECD differ by developmental stage. During infancy, the pathways between SES and ECD are explained in part by birth weight, maternal social support, stimulation activities, and learning materials. During toddlerhood the association is explained in part by child nutritional status (height‐for‐age) and learning materials. All three pathways explained some, but not all, of the SES‐ECD gaps, with family investments emerging as the largest contributor. This highlights the complex and multidimensional nature of SES‐ECD gaps, indicates that additional pathways must be integrated, and informs a nuanced understanding of the mechanisms that may explain how socioeconomic well‐being influences early thriving in a low‐resource Peruvian setting, with implications for similar Majority World contexts.

## Summary

- Socioeconomic status (SES) predicts children's health and environments in infancy and toddlerhood, but the pathways linking SES to early child development (ECD) differ by developmental stage.
- During infancy, the positive pathways between SES and ECD are explained in part by child birth weight, maternal social support, stimulation activities, and learning materials.
- During toddlerhood the association is explained in part by child nutritional status (height‐for‐age) and learning materials.
- Multiple pathways, including early life health, family well‐being, and family investment, partially explain SES disparities in ECD, underscoring the need to address both socioeconomic conditions as well as these mediating pathways.
- These findings offer insights into how socioeconomic well‐being influences early thriving in a low‐resource Peruvian setting, with implications for similar Majority World contexts.

## 1 Introduction

Socioeconomic disparities in early childhood development (ECD) have been documented worldwide across income settings (Larson et al. 2015; Letourneau et al. 2013; Schady et al. 2015). Socioeconomic status (SES) is closely linked to a range of factors that influence children's early development, including early life health, family stress, and family investment (Conger et al. 2010; Finch 2003; Hack et al. 1995). However, extant empirical research is limited in the degree to which it examines each of these paths simultaneously. It is plausible that the relative consequence of each of these pathways is different in the Majority World (i.e., low‐ and middle‐income, Global South countries) compared to the Minority World (i.e., high‐income, Global North countries) (Alam 2008), due to differences in norms, disease burden, access to services, and available resources across these settings (Henrich et al. 2010; World Health Organization 2025). For example, in Cajamarca, Peru, where the present study was conducted, geographic remoteness, extended family involvement in caregiving, and uneven access to health and educational services may shape the pathways linking SES to child development. Therefore, to design targeted ECD and parenting interventions, it is essential to identify the determinants of early thriving and better understand how socioeconomic hardship influences ECD in diverse Majority World settings.

This study examines how household SES is related to overall ECD during infancy and toddlerhood. SES refers to the social and economic resources that one has, such as assets, housing, demographic characteristics, education, income, occupation, and other indicators (Antonoplis 2023). Throughout this study, we have operationalized SES to include maternal and paternal education, material resources, and housing. We use data from a rural, northern Andean Peruvian setting to assess three key pathways to partly explain the SES association with ECD: early life health, family well‐being, and family investment. First, we estimate the direct and indirect relations between SES and ECD through these pathways using separate models for infants and toddlers. Next, we compare these two models to understand how the mechanisms underlying SES‐based ECD disparities may differ by child age. By identifying the determinants of early thriving, we hope to contribute to a deeper understanding of the mechanisms that support ECD in this context, as well as to inform the development of more targeted policies and programs.

### 1.1 SES as a Predictor of ECD

Healthy child development depends on a combination of many factors operating at varying levels in their ecological context (Bronfenbrenner and Morris 2006); these enabling environments include caregivers’ capabilities, empowered communities, supportive services, and policies (World Health Organization et al. 2018). Theories across disciplines emphasize how having higher SES can enable families to invest more—such as time, activities, financial resources—in their children's development, while being exposed to more adversity can undermine this investment and can lead to harsher, less responsive parent‐child interactions (Bradley and Corwyn 2002; Conger and Conger 2002; Conger and Donnellan 2007; Grant et al. 2003). Toxic stress, which can result from prolonged adversity without adequate adult support, can disrupt healthy brain development, with potentially lasting effects on later outcomes in learning, behavior, and physiology (Center on the Developing Child 2005; Shonkoff et al. 2012). Children from more disadvantaged backgrounds are more likely to be exposed to substandard housing, chaotic environments, family turmoil, and other stressors (Evans and Kim 2013).

Global evidence shows that lower household SES is associated with less optimal developmental outcomes for children across developmental domains (Lu et al. 2020; Richards et al. 2018). In studies across Peru, various researchers have found SES‐related gaps in children's cognitive, language, motor, social development, and physical growth development of between 0.27 and 1.40 SD (Fernald et al. 2012; Lopez Boo 2014; Ngoy et al. 2024). Importantly, these studies used a variety of measures of child outcomes, as well as conceptualizations of SES (e.g., by comparing child outcomes across wealth quintiles, levels of schooling, etc.).

### 1.2 Mechanisms Explaining the SES‐ECD Association

Although robust evidence has documented SES‐based disparities in ECD around the world, and in Peru (Fernald et al. 2012), less research has focused on the mechanisms that explain this association. A deeper understanding of these mechanisms is vital for policy‐making and achieving the Sustainable Development Goals (SDGs). In particular, SDG 3 (Good Health and Well‐being), SDG 4 (Quality Education), and SDG 10 (Reduced Inequalities) (United Nations 2026), are all relevant for ECD in different ways, and this analysis helps clarify how these factors jointly shape children's trajectories. Recent studies in Majority World settings have started to examine these mechanisms explaining SES gradients in ECD using a range of mediators (Bradley and Corwyn 2002; Rubio‐Codina et al. 2016), although this growing body of research often has been segmented by field. There is less evidence that integrates pathways across disciplines—such as public health, psychology, and economics—for a more holistic understanding of the mechanisms underlying SES gradients in ECD. This paper seeks to offer a comprehensive understanding by bringing together pathways from different academic disciplines into a single model to better understand the complex, interrelated mechanisms.

#### 1.2.1 Early Life Health Pathway

One mechanism explaining the SES‐ECD association is early life health. Research from public health emphasizes the ways in which early life health factors, such as birth weight, length‐for‐age‐z‐score (LAZ), and height‐for‐age‐z‐score (HAZ), are associated with both SES and ECD. Birth weight, often used as a proxy for pregnancy health, is linked with family SES and mothers’ education (Astone et al. 2007; Dubois and Girard 2006; Finch 2003). Low birth weight, defined as less than 2500 g at birth by the World Health Organization (WHO), is considered a risk factor for child development (Hack et al. 1995; WHO2014a).

Beyond infancy, children's HAZ is often used as a proxy for chronic malnutrition. Moderate stunting is defined as a HAZ score of less than two SD below the reference median of the WHO Child Growth Standards (WHO 2006). Low SES and maternal education are associated with stunting (Ekholuenetale et al. 2020). In turn, globally stunting has been found to be associated with lower cognitive outcomes, reduced schooling, and reduced economic productivity (Adair et al. 2013; Hoddinott et al. 2013; Walker et al. 2007; WHO 2014b).

Early life health has been found to be an important mediator between SES and ECD. In a cross‐national study which included Peru, home stimulation and LAZ explained between one‐third to one‐half of the wealth and education gradients in child development (Fernald et al. 2012). Similarly, Hamadani and colleagues’ (2014) work with children under 5 years old in rural Bangladesh found that the relationship between poverty and cognitive outcomes was largely explained by parental education, pre‐ and postnatal growth in length, and home stimulation. Finally, Rubio‐Codina and coauthors (2016) found that height‐for‐age was a mediator between SES and language outcomes in Colombia.

#### 1.2.2 Family Well‐Being Pathway

In addition to early life health, well‐being is another pathway through which SES and ECD are related. Research in sociology and developmental psychology has emphasized the important role that SES‐related stress may play in shaping child outcomes. The Family Stress Model posits that economic hardship can have a negative effect on parents’ emotions, behaviors, and relationships, and in turn their parenting strategies (Conger and Conger 2002; Conger and Donnellan 2007). In this way, economic disadvantage can exacerbate family stress, increase parents’ risk for emotional distress or internalizing disorders (e.g., depression), and negatively impact child development (Conger and Donnellan 2007). In Peru, depressive symptoms were found to be more prevalent among lower SES populations, with more access to treatment for higher SES groups (Hernández‐Vásquez et al. 2020; Villarreal‐Zegarra et al. 2025). Maternal depression is not only associated with lower SES and education levels (Field et al. 2006; Szurek‐Cabanas et al. 2024), but also child behavior problems, poorer physical growth, and lower cognitive development in some contexts (Avan et al. 2010; Liu et al. 2017; Patel et al. 2004; Surkan et al. 2012).

Another component of well‐being is social support, which is tangible or intangible assistance that a person receives or perceives (Hinson Langford et al. 1997), such as help caring for a child or emotional guidance. Evidence from the US found that higher SES was associated with more social support (Klebanov et al. 1994), and in China social support served as a mediator between SES and maternal depressive symptoms (Chen et al. 2025; Xiao et al. 2024). Importantly, social support can act as a “buffer” against stress (Cohen and Wills 1985), serving as a protective factor against maternal depression (Kim et al. 2014; Lefkovics et al. 2018) and improving mother‐child interactions (Diniz et al. 2014; Scherrer et al. 2024). Maternal social support has not only been found to relate to mother outcomes, but also to better child cognitive development and lower risk for developmental delays (Imanishi et al. 2024; Shin et al. 2019; Slykerman et al. 2005).

#### 1.2.3 Family Investment Pathway

The family investment pathway is another mechanism through which SES and ECD are related. Economists have often discussed the important ways that SES may shape parental investments in ECD, such as human and financial resources parents spend for their child's well‐being. The Family Investment Model proposes that families with higher SES have more access to financial, social, and human capital, and are therefore able to invest these resources, such as learning materials and stimulation activities, in their children's development (Conger and Donnellan 2007). In short, what parents have (including education and wealth) influences what they do with their children in regards to providing opportunities for learning, influencing how their children develop (Rao et al. 2021).

Children from higher SES households tend to be exposed to more home stimulation and learning materials (Cuartas et al. 2020; Jeong et al. 2017). Systematic reviews including Majority World settings have found that support for learning, such as learning materials and home stimulation, are consistently positively associated with ECD, even after accounting for SES (Jeong et al. 2017) (Hatch et al. In press). Across eight Majority World countries, home stimulation was a partial mediator for the relationship between caregiver education and child development (Do and McCoy 2024). In Colombia, parental education and the quality of the home environment were mediators between SES and cognitive, language, fine motor, and socio‐emotional development (Rubio‐Codina et al. 2016).

#### 1.2.4 Exploring Developmental Variation in SES‐ECD Pathways

In addition to specific pathways, different developmental periods of early childhood must be considered. Although global evidence indicates that the SES‐based disparities in ECD widen as children get older (Fernald et al. 2012), less research has focused on how these SES‐ECD associations may evolve across the distinct stages within early childhood, particularly the transition from infancy to toddlerhood. Early childhood (EC)^1^ is a period up to 8 years of age marked by rapid and dynamic changes in children's development, and treating it as a single phase can obscure important variation by developmental stage. Although many studies examine the SES‐ECD pathways during either infancy (up to 1 year), toddlerhood (1–3 years), or across the entire EC period, fewer studies explicitly compare how these pathways may differ during infancy versus toddlerhood to disentangle the specific processes that matter at different stages of development. A small but growing body of research addresses this issue. Rubio‐Codina and coauthors (2016) found that mediation between SES and ECD through parental education and the home environment occurred primarily in the 1.5–2.5‐ and 2.5–3.5‐year age groups, compared to the 0.5–1.5‐year‐old group. Hamadani and colleagues (2014) found that nutrition was an important mediator between poverty and cognitive development in the child's first 2 years, while home stimulation was important throughout the first five years.

It is also important to consider how variation in caregiving priorities can influence which pathways are key at different stages of child development. Parents’ beliefs regarding what is important for their children's development can differ across settings and influence parent‐child interactions (Bornstein 2015; Harkness et al. 2007); while some societies value infant verbal stimulation, others focus on nonverbal soothing (Richman et al. 1992). Even within the same setting, there is meaningful variability in terms of parental child development beliefs; for example, a study in rural Burkina Faso found that some parents said that children start to comprehend language in the first few months, while others said older than 2 years (Dumbaugh et al. 2023). As children develop new abilities from infancy to toddlerhood, such as talking and walking, parenting tasks change (Staples and Bates 2018). It is possible that parents prioritize child health during infancy, focusing primarily on physical growth and nutrition, whereas investment in early learning and stimulation may take more prominence during toddlerhood. Understanding how mediating pathways between SES and ECD can differ from infancy to toddlerhood requires careful consideration of distinct developmental demands across EC.

### 1.3 Context of Cajamarca, Peru

We home in on the context of Cajamarca, Peru to examine the pathways between SES and early childhood development. To provide context on the study area, Peru is characterized as an Upper‐Middle‐Income country (World Bank n.d.). Although the poverty rate in Peru has decreased significantly (60% in 2002 to 27.6% in 2024) (INEI 2025; World Bank 2025), stark differences remain in access to resources across regions (Interamerican Development Bank 2025). Evidence from Peru also highlights SES‐related differences in child development. For example, 98.6% of 2‐year‐old children from the highest wealth quintile have play materials at home compared to 85.5% from the lowest quintile (INEI and MIDIS 2025). Similarly, while enrollment in pre‐primary education is 94% (OECD 2026), children from the lowest wealth quintile, with non‐educated mothers, who are indigenous, or are considered most vulnerable are less likely to attend formal preschool, with achievement scores differing based on these preschool attendance patterns (Cueto et al. 2016). Peru has made substantial progress in reducing child undernutrition (Huicho et al. 2020). Data from the 2024 Demographic Family Health Survey (ENDES) indicates that 13.8% of children under 36 months of age suffer from chronic malnutrition (rural: 22.2%, urban: 10.1%), and 43.7% of children 6–35 months old have anemia (rural: 51.9%, urban: 40.2%) (INEI and MIDIS 2025).

Peru is ethnically and geographically diverse. This study was conducted in the Cajamarca region of Peru, in the provinces of Cajabamba, Cajamarca, and San Marcos. Cajamarca is a high‐altitude region situated in the northern Andes Mountains that is predominantly rural (64.6%) and Spanish speaking (99.5%) (INEI 2022). Despite its rich cultural traditions and natural resources, the Cajamarca region is known to be one of the most economically impoverished, with a monetary poverty rate of 45% in 2024 (Huarachi et al. 2024; INEI 2024, 2025). The principal sources of income are farming and livestock and most people live in houses with earthen floors and adobe walls (Sanchez‐Samaniego et al. 2021; Hartinger et al. 2020). Cajamarca is one of the regions with the largest increases in mean birthweight (2919 g in 2012 to 3120 g in 2019) (Carrillo‐Larco et al. 2021). Caregiving of young children is characterized by a strong emphasis on health and feeding, with mothers spending substantial time with their children and multiple caregivers are involved in childrearing (Hinckley et al. 2025).

### 1.4 The Present Study

**FIGURE 1:**
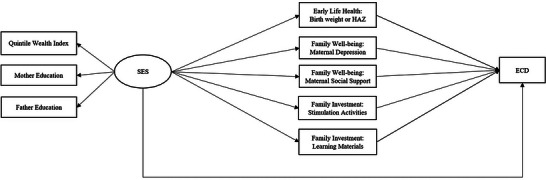
Conceptual model of the pathways between family socioeconomic status and early childhood development.

This study aims to determine how household SES directly and indirectly predicts overall ECD through the pathways of early life health, family well‐being, and family investment in both infancy and toddlerhood within a sample of children in rural, Andean Peru. We combine early life health, family well‐being, and family investment pathways. (See Figure 1 for the conceptual model.) Aim 1 is to examine the direct and indirect relations between household SES and overall ECD, modelled separately for infants aged 3–9 months old and for toddlers aged 2 years old in rural Andean Peru. Aim 2 is to describe the differences in the mechanisms through which household SES predicts overall ECD during infancy versus toddlerhood, comparing the pathways of early life health, family well‐being, and family investment. By addressing these research questions, we aim to provide a more nuanced understanding of the mechanisms that explain SES‐based disparities in ECD within a rural Peruvian setting, with implications for other similar Majority World settings.

## 2 Methods

### 2.1 Procedure and Sample

The data for this study comes from a three‐arm cluster Randomized Control Trial (RCT) in the provinces of Cajabamba, Cajamarca, and San Marcos of the rural, Andean, high‐altitude Cajamarca Region in Peru. The trial included 2702 parent‐child dyads across 164 study clusters who were enrolled when children were 3–9 months old at baseline from September 2021‐March 2023 and followed up at 2 years^2^ old at endline from September 2023‐March 2025. Children were eligible for the RCT if they did not plan to move outside the study area in the next year, did not have a diagnosed disability, and did not participate in the national *Cuna Más* home visiting program. Families were randomly assigned to one of three conditions: a digital parenting intervention (*n* = 1174), a home visiting program (*n* = 317), and a control condition (*n* = 970). In addition, a separate comparison group was also enrolled including families who were participating in *Cuna Más* (*n* = 241). Data were collected in participants’ homes by trained local assessors, including verbally‐administered questionnaires to caregivers (e.g., about child development, parenting, social support), as well as direct child assessments. See Hartinger Pena et al. 2023 for further details.

The present study includes the complete sample from the RCT (*n* = 2702). Descriptive statistics for the sample are included in Table 1. The average age of children at baseline was 5.02 months (*SD* = 1.94, range = 3–9) and at endline was 29.62 months (*SD* = 3.43, range = 24.23‐48.13). About half of the children were female (49.7%). Most caregivers who responded at baseline were mothers (97.7%), while a small proportion were fathers (2.0%).

**TABLE 1:** Sample descriptive statistics (*N* = 2702).

	*n*	% missing	%	Mean	Standard deviation	Min	Max
*Child characteristics*							
Child age (in months)—Infancy	2699	0.1%		5.02	1.94	3.00	9.00
Child age (in months)—Toddlerhood	2374	12.1%		29.62	3.43	24.23	48.13
Child sex (female)	2702	0.0%	49.70				
*Household socioeconomic characteristics*							
Maternal education (highest level completed)	2651	1.9%					
None or primary incomplete	394		14.90				
Primary complete	530		20.00				
Secondary incomplete	385		14.50				
Secondary complete	726		27.40				
Some tertiary	616		23.20				
Paternal education (highest level completed)	2324	14.0%					
None or primary incomplete	194		8.30				
Primary complete	390		16.80				
Secondary incomplete	269		11.60				
Secondary complete	894		38.50				
Some tertiary	577		24.80				
SES index	2689	0.5%					
Quintile 1 (low SES)	545		20.30				
Quintile 2	531		19.70				
Quintile 3	538		20.00				
Quintile 4	542		20.20				
Quintile 5	533		19.80				
*Measures of early life health*							
Birthweight (in kg)—Infancy	2652	1.9%		3.13	0.51	0.66	5.50
HAZ (height‐for‐age‐z‐score)—Toddlerhood	2312	14.4%		−1.56	1.02	−7.21	3.26
*Measures of family investment*							
Stimulation activities (of 6)—Infancy	2473	8.5%		3.46	1.23	0.00	6.00
Stimulation activities (of 6)—Toddlerhood	2342	13.3%		3.98	1.54	0.00	6.00
Learning materials (of 11)—Infancy	2473	8.5%		3.82	2.64	0.00	11.00
Learning materials (of 11)—Toddlerhood	2344	13.2%		6.40	2.69	0.00	11.00
*Measures of family well‐being*							
Maternal depression—Infancy	887	67.2%		2.55	3.33	0.00	21.00
Maternal depression—Toddlerhood	2276	15.8%		3.97	3.88	0.00	21.00
Maternal social support—Infancy	2192	18.9%		13.80	6.03	0.00	24.00
Maternal social support—Toddlerhood	2276	15.8%		12.96	5.59	0.00	24.00
*Early childhood development*							
Mother‐reported ECD (CREDI) raw score—Infancy	2699	0.1%		43.04	1.55	38.17	47.62
Mother‐reported ECD (CREDI) z score—Infancy	2699	0.1%		0.05	0.60	−3.37	2.27
Directly‐assessed ECD (GSED) raw score—Toddlerhood	2374	12.1%		65.82	5.80	15.15	86.64
Directly‐assessed ECD (GSED) DAZ score—Toddlerhood	2368	12.4%		−1.04	0.95	−5.95	2.64

The data and code used in this study will be made available by reasonable request to the first author. The RCT was registered at clinicaltrials.gov (*NCT05202106*). This specific study was not pre‐registered. The study was reviewed and approved by the institutional review boards of *Ethics Committee for North‐Western Switzerland*, as well as the *Comité Institucional de Ética en Investigación of Universidad Peruana Cayetano Heredia (202522)*.

### 2.2 Measures

All measures were reviewed for local relevance and analyzed to ensure reliability and validity. Below we have categorized the measures based on how they were operationalized in the models. All factors were assessed at both time points. Full details on available measures can be found in Supplemental Material 3.

#### 2.2.1 Household Socioeconomic Characteristics

We created a latent SES factor using three time‐invariant observed variables: maternal education (highest level completed, 5 categories), paternal education (highest level completed, 5 categories), and a wealth index derived via Principal Components Analysis, which was based on overcrowding, consumer durables, and housing characteristics and categorized into quintiles (Filmer and Pritchett 2001; Vyas and Kumaranayake 2006).

#### 2.2.2 Early Life Health

At baseline, early life health was operationalized as birth weight in kilograms from parent‐report or health cards. At endline, we used a height‐for‐age‐z‐score, which was measured using a standardized portable wooden stadiometer for children, manufactured in accordance with the Peruvian Ministry of Health protocols, and calculated using the “zscore06” Stata module based on WHO child growth standards (Leroy 2011).

#### 2.2.3 Family Well‐Being

At both baseline and endline, maternal depression was measured using a sum score of seven items scored on a 4‐point Likert scale from the depression subscale of the Depression, Anxiety, and Stress Scales (DASS‐21) (e.g., “I felt that I had nothing to look forward to.”) (Lovibond and Lovibond 1995). The Cronbach's alpha for maternal depression was 0.82 at baseline and 0.82 at endline. We also used a sum score of six items scored on a 5‐point Likert scale (None of the time—All of the time) from the Social Support Scale to measure availability of different sources of maternal social support, a component of well‐being, at both baseline and endline (e.g., “[Do you have] someone to take you to the doctor if you had to go?”) (Sherbourne and Stewart 1991). The Cronbach's alpha for social support was 0.71 at baseline and 0.77 at endline.

#### 2.2.4 Family Investment

We used the sum of six items from the parent‐reported Family Care Indicators (FCI) at both baseline and endline to measure parental stimulation behaviors. The FCI asks parents whether they engaged in stimulation practices (e.g., reading, counting, singing) with their child in the past 3 days, with each item scored as yes = 1 and no = 0 (Kariger et al. 2013). The FCI is conceptualized as an index of distinct caregiving behaviors. The Cronbach's alpha for stimulation activities was 0.50 at baseline and 0.61 at endline. In addition, at both baseline and endline we used a measure of learning materials available at home for young children, drawing items from International Development and Early Learning Assessment (IDELA), FCI, and additional context‐specific items (Kariger et al. 2013; Save the Children 2024). We used the sum of 11 items, scored as available in the home = 1 and not available = 0, including but not limited to children's books, movement toys, musical toys, and toys to learn about numbers, letters, and colors. The Cronbach's alpha for learning materials was 0.78 at baseline and 0.76 at endline.

#### 2.2.5 Early Childhood Development

At baseline, we used the Caregiver Reported Early Development Instruments Long Form (CREDI) as a measure of overall ECD. This caregiver‐report measures overall child development for children ages birth to 3 years old and was designed for global use (McCoy et al. 2018). The CREDI includes 108 items, from which parents respond to an age‐based subset regarding their children's development, scored as yes = 1 and no = 0 (McCoy et al. 2021). We used the developers’ scoring app to calculate the overall development raw scores (CREDI Team, n.d.). Because each child receives different items depending on their age and ability, a Cronbach's alpha for the CREDI Long Form could not be computed. At endline, we used the Global Scales for Early Development Long Form (GSED), which directly assesses development of children under 3 years old and was developed for culturally diverse and low‐resource settings. Trained and certified data collectors ask children to complete a subset of the 155 items depending on the children's age and ability (GSED Team 2019; McCray et al. 2023). We used the unidimensional overall “D‐score” (Development score) calculated using the developers’ scoring app, which is based on motor, cognitive, language, and social‐emotional tasks (WHO 2023).

#### 2.2.6 Covariates

Covariates included child age (in months) at both baseline and endline, as well as child sex assigned at birth (female vs. male).

### 2.3 Analytic Approach

We used structural equation modeling (SEM) to examine how household SES directly and indirectly predicts overall ECD. We estimated two separate partially latent structural regression models, one for infancy (children ages 3–9 months) using baseline data, and one for toddlerhood (children ages 2 years) using endline data. Estimating two separate models, rather than a single combined model, was a methodological choice to reduce model complexity and avoid potential issues with multicollinearity and model instability. Both models included a latent factor for SES as the predictor variable. The hypothesized mediators included early life health variables (birth weight for infancy, HAZ for toddlerhood), family well‐being (maternal depression and maternal social support), and family investment (stimulation activities and learning materials). The outcome was overall ECD, as measured by CREDI in infancy and the GSED in toddlerhood. For both the infancy and toddlerhood models, we controlled for child age and child sex as predictors of ECD; for the toddlerhood model, we also controlled for age‐adjusted infant overall ECD, measured by CREDI, as a predictor of toddler ECD. To account for non‐normal and missing data, we employed the robust Maximum Likelihood Robust (MLR) estimator and Full Information Maximum Likelihood (FIML), respectively (Kline 2023). See Table 1 for details on missingness. Missingness was associated with observed covariates included in the models; inclusion of a rich set of covariates helps account for potential systematic missingness, making the Missing at Random (MAR) assumption plausible and supporting the use of FIML. Although the three indicators comprising our SES latent construct are ordinal, each contains five response categories and can therefore be treated as continuous (Rhemtulla et al. 2012), further supporting the use of the MLR estimator. We assessed the adequacy of model fit using the following standards: root mean square error of approximation (RMSEA) of <0.06, confirmatory fit index (CFI) of >0.95, and Standardized Root Mean Square Residual (SRMR) of <0.08 (Hu and Bentler 1999; Kline 2023). We established whether there were differences in ECD based on SES for each age group by examining total effects. To address our first study aim, we examined the magnitude and statistical significance of the direct paths from SES to ECD, as well as the indirect paths through early life health, family well‐being, and family investment separately for the infancy and toddlerhood model. We present effects in the following order: total path (indirect and direct paths from SES to ECD), direct paths (SES to mediators, mediators to ECD, SES to ECD), and indirect paths (SES to mediator to ECD). As a sensitivity check, we conduct a multi‐group analysis to assess whether there were any differences in our results across the treatment and control groups from the original RCT. We compare a model in which structural regression paths are constrained to be equal across treatment and control groups with a model allowing only these paths to vary. These analyses focused only on the toddlerhood model, as the infancy model was estimated using data collected prior to the RCT's randomization. To address our second study aim of describing the differences in the mechanisms through which household SES predicts ECD during infancy versus toddlerhood, we first present overall model fit statistics and then descriptively compare specific pathways. All descriptive statistics were calculated using Stata/SE 18.0 (StataCorp 2025) and all structural equation models were estimated using R version 4.4.3 (R Core Team 2025) with the *lavaan* package (Rosseel 2012).

## 3 Results

### 3.1 Descriptive Statistics

Full descriptive statistics can be found in Table 1. About half (50.6%) of mothers had completed at least secondary education, while a higher proportion of fathers had completed this level (63.3%). For women, this is similar to the Peruvian secondary education completion level by 16 years old for women in the lowest SES quintile (55.1%); for men, this is similar to the middle quintile (61.0%) (INEI 2023). Based on caregiver report, the vast majority (86.1%) of children in our sample were exclusively breastfed for the first six months (or until data collection if they were younger than 6 months), which is higher than national estimates (65.9%) (MINSA 2024b). On average, children were born at 3.13 kg (*SD* = 0.51), which is slightly below the mean Peruvian birth weight in 2019 (3.26 g), yet higher than the highlands average (2954 g) (Carrillo‐Larco et al. 2021). It is important to consider that the study participants reside at high altitude, and birth weight is generally lower in higher altitude regions of Peru (Hartinger et al. 2006). In the toddler period for HAZ, children were on average 1.56 SD below the WHO reference median, and 27.4% of children were categorized as stunted—a rate notably higher than national estimates (13.8%), as well as rural areas (22.2%) and the highlands (19.6%) for children under three (INEI and MIDIS 2025; WHO 2014b). On average, stimulation activities and learning materials increased from infancy to toddlerhood, from 3.46 (*SD* = 1.23) to 3.98 (*SD* = 1.54) and 3.82 (*SD* = 2.64) to 6.40 (*SD* = 2.69), respectively. Maternal social support remained relatively constant from infancy (*M* = 13.80, *SD* = 6.03) to toddlerhood (*M* = 12.96, *SD* = 5.59), only slightly lower in toddlerhood. Most mothers were not experiencing high levels of depression during infancy nor toddlerhood (*M* = 2.55, *SD* = 3.33 and *M* = 3.97, *SD* = 3.88, respectively, range = 0–21); however, routine data checks revealed data quality issues relevant to eight specific data collectors during baseline data collection of the maternal depression measure. To minimize potential bias, we excluded 1243 cases from these data collectors, limiting the sample size for maternal depression during infancy. In infancy, children's caregiver‐reported ECD scores were, on average, slightly above the CREDI global reference populations based on the z score (*M* = 0.05, *SD* = 0.60, range = −3.37 to 2.27). However, in toddlerhood, children scored below the GSED global reference population based on the DAZ score which was directly assessed (*M* = −1.04, *SD* = 0.95, range = −5.95 to 2.64). (See Supplemental Material 2 for Correlation Matrix.)

### 3.2 Aim 1: Infancy Model

**FIGURE 2:**
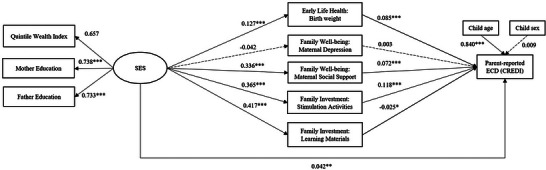
Results of the analytic model for infancy. All coefficients presented are standardized, with *p* values representing the statistical significance of the unstandardized coefficients, * *p* < 0.05. ** *p* < 0.01. *** *p* < 0.001. Dotted lines represent non‐statistically significant paths, while solid lines represent statistically significant paths. Model fit: Scaled χ^2^(38) = 370.54, *p* < 0.001; Robust CFI = 0.94; Robust RMSEA = 0.06; SRMR = 0.05. The sample size for this model was *N* = 2,699; three cases were dropped because of missingness on exogenous variables (e.g., child age). The factor loading for the Quintile Wealth Index was fixed to 1 to scale the latent SES construct. Measurement errors, residual variances, and covariances among exogenous variables were estimated but not shown in the figure.

The results from the partially latent structural regression model at baseline when children were infants are presented in Figure 2. The factor loadings for the SES latent factor can be found in Supplemental Material 1. The infancy model had acceptable overall model fit statistics, with CFI and RMSEA slightly below and above, respectively, established cutoffs: Scaled χ^2^(38) = 370.54, *p* < 0.001; Robust CFI = 0.94; Robust RMSEA = 0.06; SRMR = 0.05. The total effect of SES on ECD, including direct and indirect paths, was positive and significant (*b* = 0.179, *SE* = 0.020, *p* < 0.001, β  =  0.109). (See Table 2 for details.) The direct path from SES to ECD was positive and significant (*b* = 0.069, *SE* = 0.024, *p* < 0.01, β  =  0.042).

**TABLE 2:** Indirect and total paths from SES to early childhood development.

	Standardized coefficient	Unstandardized coefficient	*SE*
**Infancy (Baseline) model: SES → ECD (CREDI)**			
**Indirect paths**			
SES → Birth weight → ECD	0.011	0.018 ^***^	0.004
SES → Maternal Depression → ECD	0.000	0.000	0.001
SES → Maternal Social Support → ECD	0.024	0.040 ^***^	0.007
SES → Stimulation Activities → ECD	0.043	0.071 ^***^	0.008
SES → Learning Materials → ECD	−0.010	−0.017 ^*^	0.008
**Total path**			
(SES → ECD) + (SES → Mediators → ECD)	0.109	0.179 ^***^	0.020
**Toddlerhood (Endline) model: SES → ECD (GSED)**			
**Indirect paths**			
SES → HAZ → ECD	0.016	0.103 ^*^	0.043
SES → Maternal Depression → ECD	−0.002	−0.014	0.037
SES → Maternal Social Support → ECD	−0.003	−0.019	0.031
SES → Stimulation Activities → ECD	0.000	−0.002	0.071
SES → Learning Materials → ECD	0.035	0.223 ^*^	0.107
T**otal path**			
(SES → ECD) + (SES → Mediators → ECD)	0.129	0.820 ^***^	0.146

Abbreviations: CREDI, caregiver reported early development index; ECD, early childhood development; GSED, global scares of early development; SES, socioeconomic status.

* *p* < 0.05.

** *p* < 0.01.

*** *p* < 0.001.

SES is positively associated with birth weight (*b* = 0.069, *SE* = 0.01, *p* < 0.001, β  =  0.127), maternal social support (*b* = 2.178, *SE* = 0.168, *p* < 0.001, β  =  0.336), stimulation activities (*b* = 0.482, *SE* = 0.032, *p* < 0.001, β  =  0.365), and learning materials (*b* = 1.187, *SE* = 0.063, *p* < 0.001, β  =  0.417). We do not find evidence of a significant association between SES and maternal depression (*b* = −0.152, *SE* = 0.124, *p* = 0.223, β  =  −0.042).

All mediators were directly associated with ECD, with the exception of maternal depression (*b* = 0.001, *SE* = 0.008, *p* = 0.849, β  =  0.003). Birth weight (*b* = 0.256, *SE* = 0.033, *p* < 0.001, β  =  0.085), maternal social support (*b* = 0.018, *SE* = 0.003, *p* < 0.001, β  =  0.072), and stimulation activities (*b* = 0.146, *SE* = 0.014, *p* < 0.001, β  =  0.118) were each positive predictors of ECD. Learning materials had a slightly negative association with ECD (*b* = −0.015, *SE* = 0.007, *p* = 0.028, β  =  −0.025).

All indirect paths between SES and ECD are presented in Table 2. The indirect paths from SES to ECD were positive via birth weight (*b* = 0.018, *SE* = 0.004, *p* < 0.001, β  =  0.011), via maternal social support (*b* = 0.040, *SE* = 0.007, *p* < 0.001, β  =  0.024), and stimulation activities (*b* = 0.071, *SE* = 0.008, *p* < 0.001, β  =  0.043). The indirect path via learning materials was negative (*b* = −0.017, *SE* = 0.008, *p* = 0.031, β  =  −0.010) and we did not find evidence of a significant indirect path via maternal depression (*b* = 0.000, *SE* = 0.001, *p* = 0.849, β  =  0.000).

### 3.3 Aim 1: Toddlerhood Model

**FIGURE 3:**
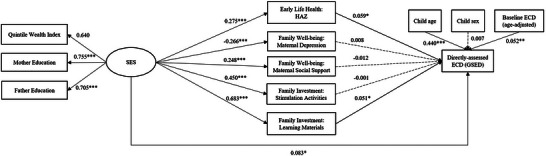
Results of the analytic model for toddlerhood. All coefficients presented are standardized, with *p* values representing the statistical significance of the unstandardized coefficients, * *p* < 0.05. ** *p* < 0.01. *** *p* < 0.001. Dotted lines represent non‐statistically significant paths, while solid lines represent statistically significant paths. Model fit: Scaled χ^2^(57) = 659.61, *p* < 0.001; Robust CFI = 0.87; Robust RMSEA = 0.07; SRMR = 0.05. The sample size for this model was *N* = 2,374; 328 cases were dropped because of missingness on exogenous variables (e.g., child age). The factor loading for the Quintile Wealth Index was fixed to 1 to scale the latent SES construct. Measurement errors, residual variances, and covariances among exogenous variables were estimated but not shown in the figure.

The results from the partially latent structural regression model at endline when children were toddlers are presented in Figure 3. The factor loadings for the SES latent factor can be found in Supplemental Material 1. The toddler model had nearly acceptable overall model fit statistics, with the exception of CFI which was further below the cutoffs: Scaled χ^2^(46) = 496.17, *p* < 0.001; Robust CFI = 0.89; Robust RMSEA = 0.07; SRMR = 0.05. The total effect of SES on ECD was positive and statistically significant (*b* = 0.820, *SE* = 0.146, *p* < 0.001, β  =  0.129). (See Table 2 for details.) The direct path from SES to ECD was positive and significant (*b* = 0.529, *SE* = 0.219, *p* = 0.016, β  =  0.083).

SES was positively associated with HAZ (*b* = 0.309, *SE* = 0.027, *p* < 0.001, β  =  0.280), maternal social support (*b* = 1.527, *SE* = 0.156, *p* < 0.001, β  =  0.248), stimulation activities (*b* = 0.762, *SE* = 0.046, *p* < 0.001, β  =  0.450), and learning materials (*b* = 2.024, *SE* = 0.079, *p* < 0.001, β  =  .683). SES is negatively associated with maternal depression (*b* = −1.138, *SE* = 0.111, *p* < 0.001, β  =  −0.266).

Only two mediators were directly associated with ECD. HAZ (*b* = 0.333, *SE* = 0.137, *p* = 0.015, β  =  0.059) and learning materials (*b* = 0.110, *SE* = 0.053, *p* = 0.038, β  =  0.051) were each positive predictors of ECD. We did not find evidence of a significant association between ECD and maternal depression (*b* = 0.012, *SE* = 0.033, *p* = 0.713, β  =  0.008), maternal social support (*b* = −0.012, *SE* = 0.020, *p* = 0.534, β  =  −0.012), nor stimulation activities (*b* = −0.003, *SE* = 0.093, *p* = 0.979, β  =  −0.001).

All indirect paths from SES to ECD for the toddler model are presented in Table 2. The indirect paths via HAZ (*b* = 0.103, *SE* = 0.043, *p* = 0.018, β  =  0.016) and learning materials (*b* = 0.223, *SE* = 0.107, *p* = 0.037, β  =  0.035) were positive. We did not find evidence of a significant path via maternal depression (*b* = −0.014, *SE* = 0.037, *p* = 0.714, β  =  −0.002), maternal social support (*b* = −0.019, *SE* = 0.031, *p* = 0.535, β  =  −0.003), nor stimulation activities (*b* = −0.002, *SE* = 0.071, *p* = 0.979, β  =  0.000).

The multi‐group analysis, which replicated the toddlerhood model across treatment and control groups, resulted in a non‐significant chi square difference test (Δχ^2^(42) = 47.418, *p* = 0.261), indicating that allowing the paths to vary by group did not significantly improve model fit. This suggests that the associations observed in toddlerhood did not differ across the treatment and control groups, indicating that results were not affected by the intervention. In addition, differences in RMSEA and CFI values between models were minimal (0.003 and 0.004, respectively).

### 3.4 Aim 2: Comparing Infancy and Toddlerhood Models

Next, we describe the differences in the mechanisms through which household SES predicts ECD during infancy versus toddlerhood. Although both models had acceptable overall model fit statistics, the infancy model had slightly better fit.

Even accounting for the measured mediating pathways, SES remains a positive direct predictor of ECD in both models; in infancy it is slightly smaller in magnitude, but more significant than in toddlerhood. Overall, we observed far greater evidence for indirect pathways in infancy relative to toddlerhood. Birth weight, maternal social support, stimulation activities, and learning materials were all found to be significant mediators during infancy. In contrast, during toddlerhood, we only found evidence of HAZ and learning materials to be significant mediators. Maternal depression was not found to be a significant mediator in either model.

Similarly, variance decomposition analysis revealed differing patterns in infancy versus toddlerhood (See Table 3). During infancy, most of the SES‐ECD association was indirect (62%) (through mediating pathways), while only 38% was direct. The largest part of the SES‐ECD indirect effect in infancy was via the family investment pathway (30%), followed by family well‐being (22%), and then early life health (10%). In contrast, during toddlerhood, most of the SES‐ECD effect was direct (64%), with only 36% as indirect. Although competing mediation (i.e., a small, negative indirect path via family well‐being) complexifies the specific interpretation of the indirect pathways, variance decomposition again points to the family investment pathway being the strongest of the three during toddlerhood.

**TABLE 3:** Variance decomposition through mediating pathways.

	Percentage of total variance explained
**Infancy (Baseline) Model: SES → ECD (CREDI)**	
**Direct**	38%
**Indirect**	62%
Early life health pathway	10%
Birth weight	
Family well‐being pathway	22%
Maternal depression	
Maternal social support	
Family investment pathway	30%
Stimulation activities	
Learning materials	
**Toddlerhood (endline) model: SES → ECD (GSED)**	
**Direct**	64%
**Indirect**	36%
Early life health pathway	12%
HAZ	
Family well‐being pathway	−4%
Maternal depression	
Maternal social support	
Family investment pathway	27%
Stimulation activities	
Learning materials	

Abbreviations: CREDI, caregiver reported early development index; ECD, early childhood development; GSED, global scares of early development; SES, socioeconomic status.

## 4 Discussion

Although SES gradients in ECD are well‐documented worldwide, research on the mechanisms underlying SES‐based disparities in child development has been limited in its integration across fields and in diverse settings. Our study addresses this gap by drawing on frameworks from different academic disciplines to examine and compare multiple pathways simultaneously in two distinct periods of EC. Specifically, we examined how SES predicts ECD directly, and via early life health, family well‐being, and family investment in a sample of 2702 parent‐child dyads from northern Andean Peru. We then compared these pathways across infancy and toddlerhood to understand potential differences in SES‐based ECD disparities. Our results suggest important implications for timing of specific ECD interventions. We found SES‐based ECD disparities exist as early as 5 months in our sample. Although SES matters for predicting children's health and environments across EC, the role that different hypothesized mediators play in explaining SES‐based ECD disparities differed by developmental stage. During infancy, SES‐ECD disparities appeared to relate to children's health, how much social support their mother received, and the learning environment to which they were exposed—generating new evidence for SDG 3 (Good Health and Well‐Being) and SDG 4 (Quality Education). In contrast, children's nutritional status (i.e., HAZ) and learning materials they had at home explained disparities in toddlerhood. During both infancy and toddlerhood, the family investment mediating pathway explains more variance in the SES‐ECD link than the family well‐being and early life health pathways.

### 4.1 Persistent SES‐ECD Associations

Considering the total effects, children with higher SES tended to have better ECD, both as infants and toddlers. The magnitude of this relationship was larger in toddlerhood than infancy, as expected based on research on the SES‐ECD gap widening with age (Black et al. 2017; Fernald et al. 2012). Even after accounting for the hypothesized mediating pathways and control variables, SES remained directly associated with ECD across EC, suggesting that there may be additional SES‐ECD pathways in this setting that were not captured in our model. This persistent association underscores that SES remains an important driver of ECD across the EC period, highlighting the importance of SDG 10 (Reducing Inequalities) and aligning with prior Peruvian research showing that the SES gradient persists even after accounting for key mediators, such as preschool, nutrition, and years of schooling, home stimulation, and LAZ (Fernald et al. 2012; Lopez Boo 2014).

### 4.2 SES Disparities in Mediators

Across EC, clear SES disparities are evident in all three hypothesized mediating pathways (early life health, family well‐being, and family investment). During infancy, children from higher SES tend to have higher birthweight, more maternal social support, be engaged in more stimulation activities, and have more access to learning materials. During toddlerhood, children from higher SES have higher height‐for‐age scores, more maternal social support, less maternal depression, are engaged in more stimulation activities, and have more access to learning materials. The consistency of these associations between SES and the mediators during both developmental stages suggests that SES matters for predicting children's early experiences that may account for SES‐disparities in ECD across EC. Notably, the magnitude of the relationships between SES and these mediators was stronger in toddlerhood than infancy for most pathways.

Maternal depression was the only SES–mediator association that showed differing patterns between infancy and toddlerhood. We only found evidence that lower SES predicts higher maternal depression during toddlerhood, not infancy. This finding reflects mixed evidence in the literature regarding when maternal depression seems to be more pronounced throughout the early childhood period; one study in France found higher maternal depressive symptoms when children were toddlers compared to infants, while another study in Australia found higher depressive symptoms for mothers of infants compared to toddlers (Giallo et al. 2015; van der Waerden et al. 2015). Also, this finding may reflect a progressive accumulation of psychological strain that becomes more visible as caregiving demands shift when children become more mobile, develop language, and require more behavioral regulation support.

### 4.3 Developmental Differences in Mediator‐ECD Associations

Although SES consistently predicts disparities across mediating pathways relatively consistently across developmental stages, the associations between mediators and ECD vary between infancy and toddlerhood.

During infancy, almost all mediators predicted improved ECD; higher birthweight, more maternal social support, and more stimulation activities translated into better ECD. Surprisingly, we also found that more learning materials was associated with lower ECD scores. However, this negative correlation was small and only statistically significant when accounting for other mediators. We interpret this to mean that above and beyond stimulation and other factors, having access to toys does not support ECD, affirming that parenting behaviors may be more important and direct contributors to children's development than mere availability of materials (Koşkulu et al. 2021). More research is needed to confirm this hypothesis, and to explore potential interactive relations between materials, beliefs, practices, and so forth.

In contrast, during toddlerhood only higher height‐for‐age and more learning materials were associated with improved ECD scores. The learning materials association is consistent with prior literature in the Majority World where books in the home, an aspect of learning materials, was found to mediate the relationship between caregiver education and child development (Jeong et al. 2017). The lack of significant association between stimulation activities and ECD in toddlerhood is surprising, especially in light of a recent meta‐analysis which found positive links between these constructs (Hatch et al. In press) and evidence from Majority World settings in which stimulation activities mediated the relationship between caregiver education and child development in samples of children 0–36 months and 3–4 year olds (Do and McCoy 2024; Jeong et al. 2017).

The stronger link between mediators to ECD in infancy than toddlerhood may relate to local caregiving practices. In particular, in our study setting a lot of attention and care is paid to children under 2 years of age and mothers tend to carry young children on their backs while they complete domestic and agricultural activities (Ames 2013). As children get older and spend less time in such close physical contact with their mother, they may have more interactions with other caregivers or siblings which can influence their development.

Maternal depression does not translate to differences in ECD outcomes in neither infancy nor toddlerhood. Although this was surprising in light of prior studies that have found maternal depression associated with child development (Liu et al. 2017; Patel et al. 2004), it is in line with work in Peru that did not find evidence of an association between maternal depression and toddler ECD (Alvarado et al. forthcoming).

### 4.4 Developmental Differences in Indirect SES‐ECD Pathways

Overall, our results indicate that early life health, family well‐being, and family investment are key pathways through which SES influences ECD, although they operate distinctly across different developmental stages. Our evidence provides support for the role of physical health as a pathway between SES and ECD, as well as the theorized models of family stress and investment (Conger and Conger 2002; Conger and Donnellan 2007). However, we observed more significant indirect pathways in infancy than toddlerhood.

During infancy, the SES‐ECD pathways via birthweight, maternal social support, and stimulation activities were positive. The birthweight finding aligns with work in Bangladesh which found that birth size and growth in the first 2 years were important mediators of the effect of poverty on children's cognition (Hamadani et al. 2014), and is consistent with the sensitive period for stunting to predict cognitive and executive functioning outcomes in the prenatal period through 2 years of age (Black et al. 2017). The positive social support pathway is consistent with evidence from mothers within their first year postpartum, where social support was positively correlated with infant care, breastfeeding, maternal adaptation (Ni and Siew Lin 2011), as well as other studies in Peru that have found relying on family members for social support to be key in this setting (Kim et al. 2019; Hinckley et al. 2025).

During toddlerhood, only two indirect pathways between SES and ECD emerged: HAZ and learning materials. Higher SES is associated with better child health, which in turn translates to better ECD, supporting prior public health work that early SES‐based disparities in health are meaningful for children's physical development (Ekholuenetale et al. 2020), and aligning with evidence that these health disparities are also important for broader developmental outcomes (Hamadani et al. 2014; Rubio‐Codina et al. 2016). Importantly, this early life and nutrition health pathway is one that Peru's social programs are already targeting. For example, the “*Juntos*” conditional cash transfer program targets families living in poverty with a child or pregnant mother and requires them to attend prenatal care visits and early child growth and development checkups (Silva Huerta and Stampini 2018). Similarly, the “*Cuna Más*” urban daycare program for families living in poverty provides iron‐rich complementary feeding to reduce anemia (Tarazona‐Meza et al. 2025). The learning material pathway suggests that children from higher SES families tend to have more learning materials and better ECD. Our finding that learning materials, but not stimulation activities, mediated the SES‐ECD relationship aligns with prior work; evidence from 14 Majority World countries with children around 8 years old found that the presence of home reading materials, but not reading behaviors, mediated the link between SES and early literacy outcomes (Zuilkowski et al. 2019).

We did not find evidence that maternal depression mediates the SES‐ECD link across EC. This may reflect limitations in how maternal mental health was measured, including the possibility that maternal depression was underestimated or not fully captured due to cultural norms and limited experience in discussing mental health in this context. Descriptive analysis of maternal depression levels revealed that most mothers were not experiencing high levels of depression; however, there was variability in this measure, suggesting that the findings were not solely due to truncated variance in the measure. This highlights the need to explore additional measures of mental health that capture more variability in symptoms, such as measures tailored to the cultural context. It is also possible that the effect of maternal depression on early childhood outcomes is buffered in a context involving multiple caregivers because children are also interacting with other caregivers and siblings. Another hypothesis is that mothers with depressive symptoms may receive increased social support, serving as a protective factor. This highlights the need for future research to better understand how maternal depression evolves during the early childhood period and shapes child outcomes.

Early life health and learning materials were the only two pathways that showed constant influence across developmental stages. Our findings suggest that the mechanisms through which SES relates to ECD differ by developmental stage, consistent with Hamadani's results (Hamadani et al. 2014). Although almost two‐thirds (62%) of the SES‐ECD association was explained by the mediating pathways in infancy, the opposite was true in toddlerhood (only 36% explained by mediators). These findings suggest that the mechanisms included in the family investment, family well‐being, and early life pathways do a generally good job of explaining SES‐ECD gaps in infancy. However, our results suggest that in toddlerhood, other mechanisms unaccounted for in this model (e.g., interactions with other caregivers, access to daycare services, the emergence of discipline practices) may become more salient. Infants tend to spend substantial time in close physical contact with their mothers so mother‐factors may be particularly salient in infancy, but may change as child mobility increases or new siblings are born. Another potentially relevant factor is feeding practices. The Peruvian Ministry of Health promotes exclusive breastfeeding until 6 months old then complimentary feeding until 2 years or more (MINSA 2024a), and the rate of breastfeeding in our sample is extremely high. We did not include breastfeeding as a mediator due to its lack of variation. It is possible that the transition from exclusive breastfeeding to complimentary feeding may be a period where SES‐related differences in diet quality and nutrition may emerge. Beyond developmental and cultural potential explanations, differences in how the outcome of ECD was measured could also contribute to the variation in the observed pathways. In infancy, ECD was caregiver‐reported, while in toddlerhood it was directly‐assessed. Therefore, the stronger associations we saw between caregiver‐reported mediators and caregiver‐reported ECD in infancy could be an artifact of same‐reporter bias.

### 4.5 Multidimensional Pathways Linking SES and ECD

The family investment pathway (including stimulation activities and learning materials) emerged as a consistent and substantial contributor to the SES‐ECD association across both infancy and toddlerhood, accounting for nearly one‐third (27%–30%) of the observed SES‐driven gaps in children's ECD outcomes. These findings affirm prior work showing that family investments may be a particularly powerful way that SES shapes children's ECD outcomes in early childhood (e.g., Coley et al. 2025).

At the same time, while the family investment pathway explains the largest share of the SES‐ECD gaps, it does not account for them fully. Early life health and family well‐being pathways also explained part of the SES‐ECD link, though to a lesser extent (at most 22%). Taken together, these findings suggest that SES disparities in ECD emerge through multiple mechanisms, rather than any single pathway alone. Evidence that all three pathways contribute to the SES‐ECD gaps highlights their multidimensional nature and suggests that inequities emerge based on a complex set of factors.

However, even when considered jointly, these three pathways only explain part of the SES‐ECD association. This finding highlights the complexity of SES‐ECD links. No single theorized pathway accounted for all—or even most—of the observed inequalities in ECD outcomes. This complexity was particularly apparent in toddlerhood, when children likely rely on a more complex set of ecological inputs to support their ongoing development (Bornstein and Tamis‐LeMonda 2010). The mediating pathways in our toddlerhood model explained substantially less of the SES‐ECD link, suggesting that alternative pathways not included in our data (e.g., the role of expanding networks of caregivers, access to ECCE) that may play an increasingly important role during this developmental period need to be considered.

Overall, our findings suggest that SES gaps in ECD are best understood through a multidimensional lens that integrates theories from different academic disciplines. The family investment, family well‐being, and early life health pathways each capture important, yet incomplete, aspects of the processes through which SES shapes ECD. Integrating these perspectives, while also identifying additional pathways that may explain remaining inequalities, is essential for advancing our theoretical understanding of SES‐ECD gaps.

The multidimensional and complex nature of SES‐ECD links has important implications for policy and intervention, providing support for the holistic approaches to intervention as a means of meeting SDG 10 (e.g., the NCF; World Health Organization et al. 2018). First, our findings highlight the importance of addressing disparities in family investments, which emerged as the largest contributor to SES‐related Gaps in ECD. Interventions that strengthen family investments (e.g., stimulation‐focused parenting programs, such as home visiting; Britto et al. 2017; Jeong et al. 2021), may represent particularly promising strategies to reduce inequities. At the same time, because SES disparities arise through multiple pathways, it is essential to complement family investment interventions by also pursuing other approaches that combat inequities more directly (e.g., cash transfer programs) and via other pathways (e.g., stress, child health) to advance progress toward SDG 10.

### 4.6 Limitations and Future Directions

We acknowledge the present study has several limitations. First, we are not able to establish temporal precedence for the estimated models as the mediators and outcome are measured at the same time point. Future research should employ data collection methods to assess causal pathways (e.g., longitudinal and/or experimental designs). Second, while the reliability statistics for most measures were adequate, the Cronbach's alpha for stimulation activities at baseline was lower than desired (0.50), likely reflecting its nature as a composite of dichotomous indicators (Sun et al. 2007). Although the FCI provides information on whether stimulation was provided, future research would benefit from incorporating measures that assess the frequency and qualitative aspects of stimulation. Third, while both models include the same theorized pathways and outcomes, the outcome measures and method for operationalizing the early life health pathway are different; therefore, models are not directly comparable. Fourth, our measure of maternal depression was limited by substantial missing data and may not have fully captured variability in symptoms, suggesting future research would benefit from more sensitive and culturally appropriate measures of caregiver mental health. Fifth, although the measure of learning materials included a wide range of items, it did not include all possible materials that children use to learn and develop. It was operationalized as a sum score and did not differentiate between types of learning materials that may differentially relate to child development outcomes. Therefore, future research using more comprehensive and nuanced measures of different types of learning materials is needed to unpack this unexpected finding. Finally, because all measures are caregiver‐reported in the infancy model, same reporter bias could have inflated the associations. Future research should consider using consistent measurement approaches—either both directly‐assessed or both caregiver‐reported—at both time points to avoid this potential bias.

### 4.7 Conclusions and Implications

The present study examines how household SES predicts ECD in a rural, Andean Peru. We advance the literature by integrating multiple pathways—early life health, family well‐being, and family investment—that are traditionally examined separately across different academic disciplines. Our study is situated in a Majority World setting that remains underrepresented in the literature on SES‐ECD pathways. Overall, our findings highlight that SES exerts influence on ECD through multiple pathways, and that the relative importance of these mechanisms differs between infancy and toddlerhood. Importantly, while the family investment pathway explained the largest share of SES‐ECD disparities, all three pathways contributed to these gaps; this highlights the complex and multidimensional nature of SES‐ECD gaps. These results underscore the need for more comprehensive approaches to combatting SES disparities in ECD, in line with the SDGs’ interdisciplinary approach. Addressing these inequalities will require not only improving families’ socioeconomic conditions directly, but also prioritizing interventions that strengthen family investments and simultaneously addressing disparities through other pathways through which SES influences children, such as child health, family well‐being, and social support interventions.

## Author Contributions

**Lena Jäggi**: conceptualization, writing – review and editing, methodology, supervision, data curation. **Milagros Alvarado**: writing – review and editing, investigation. **Andreana Castellanos**: writing – review and editing. **Maria Catalina Gastiaburu Cabello**: writing – review and editing, investigation. **Dana Charles Mccoy**: conceptualization, writing – original draft, methodology, writing – review and editing, supervision. **Daniel Mäusezahl**: funding acquisition, writing – review and editing, supervision, resources. **Sarah Hatch**: writing – review and editing. **Günther Fink**: writing – review and editing, conceptualization, funding acquisition, methodology, supervision, resources. **Kristen Hinckley**: conceptualization, investigation, writing – original draft, methodology, writing – review and editing, formal analysis, data curation. **Leonel Aguilar**: writing – review and editing. **Stella M. Hartinger**: supervision, resources, writing – review and editing.

## Funding

This project was funded by the Basel Research Centre for Child Health.

## Ethics Statement

This study was approved by the appropriate Ethics Committee for North‐Western Switzerland, as well as the Comité Institucional de Ética en Investigación of Universidad Peruana Cayetano Heredia (202522) Ethics Boards. No material has been reproduced from other sources.

## Conflicts of Interest

We have no conflicts of interest to disclose.

## Supporting information




**Supporting information**: desc70260‐supp‐0001‐SuppMat.docx

## Data Availability

The data and code used in this study will be made available by reasonable request to the first author.

## Endnotes

1 Throughout the manuscript, when we mention “EC”, we are referring to the early childhood period that includes both infancy and toddlerhood.

2 Throughout the manuscript we refer to endline data collection for toddlers as 2 years because this is the majority (*n* = 2,232). However, some children were 3 years old (*n* = 141), and one child was 4 years old.
