# Supplementary material for: Pathways Between Family Socioeconomic Status and Early Childhood Development: Evidence From Andean Peru
Source: Dev Sci. 2026 Aug 18;29(5):e70260. doi: 10.1111/desc.70260 (PMC13487364; doi:10.1111/desc.70260)
Supplement: Supplementary file 1 [file DESC-29-e70260-s001.docx]

# Pathways between Family Socioeconomic Status and Early Childhood Development: Evidence from Andean Peru

## Supplemental Material 1.

| *SES Latent Factor Loadings in Infant and Toddler Models* | | |
| --- | --- | --- |
|  |  |  |
|  | Standardized Loading | Unstandardized Loading (SE) |
| **Infancy Model: SES Latent Factor** |  |  |
| Quintile SES Index | 0.657 | 1 (0) |
| Maternal Education | 0.738 | 1.313 (0.053) |
| Paternal Education | 0.733 | 0.992 (0.039) |
| **Toddlerhood Model: SES Latent Factor** |  |  |
| Quintile SES Index | 0.640 | 1 (0) |
| Maternal Education | 0.755 | 1.376 (0.051) |
| Paternal Education | 0.705 | 0.988 (0.037) |
| Notes: SES = Socioeconomic Status, *** p < .001. The factor loading for the Quintile Wealth Index was fixed to 1 to scale the latent SES construct. | | |

## Supplemental Material 2.

## Supplemental Material 3.

Measure: DASS-21 (Depression, Anxiety, and Stress Scales) – depression subscale (Lovibond & Lovibond, 1995)

Response options:

Does not apply to me at all

Applied to me to some degree or some of the time

Applied to me to a considerable degree or most of the time

Applied to me very much or most of the time

You will read 21 statements about how you felt over the last week. Please choose how much the statement applied to you over the past week. There are no right or wrong answers. Do not spend too much time on any statement.

1. I could not seem to experience any positive feelings at all
2. I found it difficult to work up the initiative to do things
3. I felt that I had nothing to look forward to
4. I felt down-hearted and blue
5. I was unable to become enthusiastic about anything
6. I felt I was not worth much as a person
7. I felt that life was meaningless

Measure: Social Support Scale

This measure is under copyright, but full details can be found at the following link: <https://www.rand.org/health/surveys/mos/social-support/survey-instrument.html>.

Measure: Parental Stimulation Behaviors - Family Care Indicators (Kariger et al., 2013)

In the past 3 days, did you or anyone in the household 15 years or older participate in any of the following activities with (child):

1. Read books to (child) or look at pictures in a book with (child)?
2. Tell stories to (child)?
3. Sing songs to (child) or with (child), including lullabies?
4. Take (child) for a walk outside the house?
5. Play with (child)?
6. Name, count, or draw objects with (child)?

Measure: Learning Materials

Some of the items in this measure are under copyright, limiting our ability to provide the full measure details here.
